# Preparation and Characterization of Polymer Composite Materials Based on PLA/TiO_2_ for Antibacterial Packaging

**DOI:** 10.3390/polym10121365

**Published:** 2018-12-09

**Authors:** Edwin A. Segura González, Dania Olmos, Miguel Ángel Lorente, Itziar Vélaz, Javier González-Benito

**Affiliations:** 1 Universidad Interamericana de Panamá, Research Direction (DI-UIP 6338000), Av. Ricardo J. Alfaro, Panama City, Panama; edwin_segura@uip.edu.pa; 2 Department of Materials Science and Engineering and Chemical Engineering, Instituto de Química y Materiales Álvaro Alonso Barba (IQMAA), Universidad Carlos III de Madrid, Leganés 28911, Madrid, Spain; malorente@ing.uc3m.es; 3 Departamento de Química, Facultad de Ciencias, Universidad de Navarra, 31080 Pamplona, Navarra, Spain; itzvelaz@unav.es

**Keywords:** Polylactic acid (PLA), TiO_2_ nanoparticles, polymer nanocomposites, antibacterial packaging

## Abstract

Polymer composite materials based on polylactic acid (PLA) filled with titanium dioxide (TiO_2_) nanoparticles were prepared. The aim of this work was to investigate the antibacterial action of TiO_2_ against a strain of *E. coli* (DH5α) to obtain information on their potential uses in food and agro-alimentary industry. PLA/TiO_2_ systems were prepared by a two-step process: Solvent casting followed by a hot-pressing step. Characterization was done as a function of particle size (21 nm and <100 nm) and particle content (0%, 1%, 5%, 10%, and 20%, wt %). Structural characterization carried out by X-ray diffraction (XRD) and Fourier Transformed Infrared spectroscopy (FTIR) did not reveal significant changes in polymer structure due to the presence of TiO_2_ nanoparticles. Thermal characterization indicated that thermal transitions, measured by differential scanning calorimetry (DSC), did not vary, irrespective of size or content, whereas thermogravimetric analysis (TGA) revealed a slight increase in the temperature of degradation with particle content. Bacterial growth and biofilm formation on the surface of the composites against DH5α *Escherichia coli* was studied. Results suggested that the presence of TiO_2_ nanoparticles decreases the amount of extracellular polymeric substance (EPS) and limits bacterial growth. The inhibition distances estimated with the Kirby-Bauer were doubled when 1% TiO_2_ nanoparticles were introduced in PLA, though no significant differences were obtained for higher contents in TiO_2_ NPs.

## 1. Introduction

Nowadays, the impact of plastic waste is a worldwide concern in our society. Consequently, the research on biodegradable materials is a response to a global need. Polylactic acid (PLA), (–[CH–(CH_3_)–COO]_n_–), belongs to the family of aliphatic polyesters and is an environmentally friendly polymer that has been widely used for producing biodegradable, biocompatible, and compostable materials [1,2]. PLA is a thermoplastic polymer with tunable mechanical properties (depending on the crystalline to amorphous fractions) that, thanks to its biodegradability, it can replace other non-degradable polymers in several applications in such a way that it improves the environmental side-effects of non-degradable polymers. PLA is present in many different applications. For example, in biomedical industry is used for sutures, films, implants, or scaffolds for tissue engineering applications [3,4]. In agro-alimentary industry, PLA is present in food packaging containers, in the manufacture of greenhouse films, or for biodegradable yard-waste bags [5,6].

In the field of agro-alimentary industry, for example for food packaging applications, apart from the biodegradable character of the material, it would be worth producing materials that inhibit bacterial growth and biofilm development. Biofilms are resistant to many antibiotics and other antimicrobial agents. To avoid or to reduce the possible degradation of PLA, one alternative could be the incorporation of some additives such as TiO_2_ nanoparticles (NPs). The interest in TiO_2_ nanoparticles is twofold. First, TiO_2_ NPs are able to absorb most of the UV radiation, thus preventing polymer degradation from environmental aging due to the exposure of plastics to UV light. Secondly, TiO_2_ inhibits to certain extent bacterial growth. Therefore, the introduction of TiO_2_ nanoparticles may be an efficient tool, not only for modifying some polymer properties, but also for hindering its degradation from bacteria or from environmental aging in outdoor materials.

Titanium dioxide, TiO_2_, has attracted the interest in many fields for its photocatalytic and bacteriostatic activity [7]. In the presence of light, TiO_2_ is able to produce the transition of an electron towards the conductive band favoring the oxidative capacity of other species by generating active agents like radicals. Keeping in mind that bacteria, when subjected to oxidative stress, are able to unleash a specific self-destruction mechanism; it is easy to understand that TiO_2_ is a material with bactericide activity which behavior is enhanced in the presence of light. Apart from the well-known bactericidal properties of TiO_2_ [8,9,10,11,12,13,14,15], as well as the fact that its biocompatibility and small size when TiO_2_ is used in the form of nanoparticles, it improves the catalytic effect of such materials [16,17,18,19], having a great potential in applications related with environment purification, decomposition of carbonic acid gas into hydrogen gas, etc. This filler is usually applied as pigment, adsorbent, catalyzer support, filter, coatings, and dielectric materials. Moreover, due to its bacteriostatic behavior, the TiO_2_ is also useful for the inhibition of odors and can be part of a self-cleaning system for specific surfaces. Such advantages make the TiO_2_ an inorganic filler ideal for the development of nanocomposite materials resistant to UV radiation, probably resistant to thermal degradation, and may inhibit the formation of harmful biofilms. For that reason, the addition of TiO_2_ nanoparticles could provide the final material some of the functional properties of the proper TiO_2_ like the UV radiation protection and the bactericidal activity.

Apart from the functional properties that the filler itself confers the final material, in most polymer nanocomposite materials, particle size is an important factor affecting the final behavior of composite materials and influencing the physical properties of the material [20]. For the same amount of particles, the ones with a smaller size would provide a larger surface to be in contact with the polymeric matrix and, for that reason; a larger interfacial region is formed. This effect is explained in detail in a recent article by J. Gonzalez-Benito et al. [21] in which the influence of TiO_2_ particles size in the thermal expansion coefficient of nanocomposites materials based on a poly(ethylene-*co*-vinylacetate)matrix, was studied.

In this work polymer nanocomposites based on PLA/TiO_2_ were prepared and characterized. TiO_2_ nanoparticles with different particle sizes, 21 nm and <100 nm, were selected. The nanoparticles were mixed and dispersed in the PLA matrix by solvent casting and the final materials were obtained after hot pressing the casted films. The effect of particle size and content in structural and thermal properties and materials behavior against bacterial growth and biofilm development of a strain of *E. coli* (DH5α) was investigated.

## 2. Experimental

### 2.1. Materials

Polylactic acid (PLA) was provided by Resinex Spain, SL, and manufactured by Nature Works LLC (Blair, NE, USA) (Ref. code: PLA Polymer 7032D; glass transition temperature, *T*_g_ = 55–60 °C; melting temperature, *T*_m_ = 160 °C; and processing temperature 200–220 °C). To prepare the nanocomposites, TiO_2_ nanoparticles with two different particle sizes, 21 nm and <100 nm, were used (Sigma Aldrich, St. Louis, MO, USA, with reference numbers 718467, and 634662, respectively). The solvent used to prepare the polymer solutions and particles suspensions was dichloromethane (purity 99.9%, Sigma Aldrich).

### 2.2. Sample Preparation

**Figure 1:**
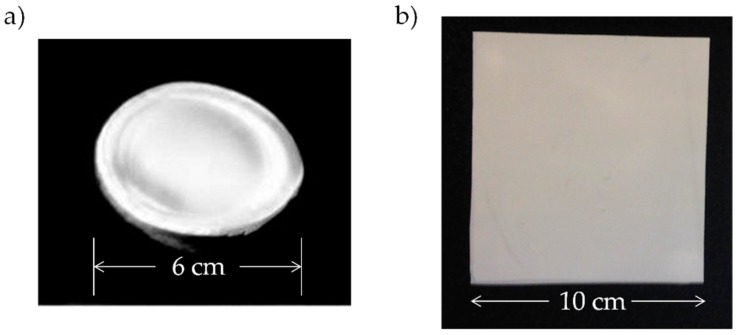
Example of a pre-film of PLA/TiO_2_ composite obtained (**a**) after casting and (**b**) after the hot-pressing step.

PLA/TiO_2_ nanocomposites films were prepared with different particle content (0%, 1%, 5%, 10%, and 20% weight percentages, wt %). Films of ca. 2.0 g were obtained according to the protocol described in Reference [22]. A solution of 10% wt/vol of PLA in dichloromethane (CH_2_Cl_2_) was mixed with a suspension of TiO_2_ NPs in CH_2_Cl_2_ (previously sonicated). This mixture (PLA+TiO_2_ in CH_2_Cl_2_) is stirred for 1 h at room temperature and then casted on a Petri dish (φ = 60 mm) to obtain pre-films. After drying at 40 °C for 24 h, the pre-films were placed between two Kapton® sheets inside a hot plate press (FONTIJE PRESSES, TP400 model, Fontijne Presses, Barendrecht, The Netherlands) and processed at 30 kN and 160 °C for 10 min, obtaining 10 × 10 cm^2^ films with an average thickness of 200 μm. The prepared films were stored in a desiccator. In Figure 1, an example of a pre-film obtained after casting and the corresponding film obtained after the hot-pressed step are shown.

Sample labeling follows the notation: PLA/TiO_2_-Particle size-particle content, in weight percentage. For example, a sample with the following code: PLA/TiO_2_-21-5 refers to a composite of PLA filled with TiO_2_ nanoparticles with a particle size of 21 nm and a particle content of 5% by weight.

### 2.3. Characterization Techniques

Structural characterization of the samples was done by X-ray diffraction (XRD) and Fourier Transformed Infrared Spectroscopy (FTIR). X-Ray diffraction experiments were done in a Phillips X’Pert X ray diffractometer (Malvern Panalytical Ltd, Malvern, UK) in the range 2θ = 3–70° using the K_α1_ radiation from copper with a wavelength, λ = 0.15406 nm. The working conditions were set at 40 kV and 40 mA. Fourier transformed infrared (FTIR) spectra were recorded using an FTIR Nicolette Avatar 360 (Analytical Instruments Brokers LLC, Minneapolis, MN, USA) equipped with a Golden Gate ATR accessory (diamond window), from 600 to 4000 cm^−1^ with a resolution of 2 cm^−1^ and averaging 32 scans, at room temperature with the software OMNIC ESP v5.1 (ThermoFisher Scientific Inc., Waltham, MA USA).

Thermal characterization of the materials was done using differential scanning calorimetry (DSC) and thermogravimetric analysis (TGA). DSC experiments were carried out in a Mettler Toledo DSC822^e^ instrument (Mettler Toledo, Greifensee, Switzerland). The thermal cycle was: (i) heating scan from 40 to 200 °C at 100 °C ·min^−1^; (ii) 5 min at 200 °C; (iii) cooling scan from 200 to 10 °C at 20 °C ·min^−1^; (iv) 5 min at 10 °C; and (v) a final heating scan from 10 to 200 °C at 20 °C ·min^−1^. Thermal transitions of the polymer, glass transition, melting and crystallization were analyzed from the heating and cooling scans. Thermal degradation of the samples was studied by TGA. The experiments were done in a TGA-SDTA 851 Mettler Toledo (Mettler Toledo, Greifensee, Switzerland) from 30 to 750 °C at a heating rate of 10 °C·min^−1^ under a nitrogen atmosphere (gas flow of 20 mL·min^−1^).

### 2.4. Biofilm Development and Bacterial Growth

The behavior of the materials against bacterial growth and biofilm development was studied using a strain of *Escherichia coli* (DH5α) following two different approaches. First, bacterial cultures on the surface of the films were done and biofilm development was studied. To do these experiments, an aliquot of the *E. coli* strain was heated and 90 µL of bacteria were mixed with 2910 µL Luria Bertani media (LB). The mixture was stirred at 200 rpm and incubated for 12 h at 37 °C. After that, the suspension was diluted 1/100 to a final volume of 20 mL. Cultures on the surfaces of the PLA/TiO_2_ materials were grown in a 24 microwell plate (ThermoFischer Scientific, Waltham, MA, USA) using the DH5α *E. coli* strain. Square samples of approximately 0.5 cm^2^ were glued on stainless steel discs with a diameter of 10 mm using cyanoacrylate-based glue. The samples were sterilized with a 70% (wt %) solution of ethanol directly sprayed on the surface and dried in a laminar flow hood. All the processes were done in a sterile environment. Then, 1 mL of the 1/100 dilution previously prepared was added to each well plate and incubated in aerobic conditions for 3 h at 37 °C with a continuous agitation at 150 rpm. After incubation, the LB medium containing the bacteria was removed from the multiwell plate and rinsed using 1 mL of physiological saline solution (NaCl 0.9% wt) to eliminate the poorly adhered cells from the surface of the materials. For examining biofilm growth on the surface of the PLA/TiO_2_ systems, a scanning electron microscope, SEM, Philips XL30 (FEI Europe Ltd., Eindhoven, The Netherlands) was used. Micrographs at different magnifications (50×, 5000× and 8000×) were collected. The voltage was set at 10 kV and the working distance at ~10 mm. To avoid charge accumulation, the samples were gold coated by sputter deposition.

The second approach to study the antibacterial behavior of the nanocomposites consists of a modification of the Kirby-Bauer diffusion test [23,24] following the protocol described in a previous work [25]. For these studies, a seed of the *E. coli*, DH5α competent cells, from ThermoFischer Scientific (Waltham, MA, USA) was used. In this case, 100 μL of bacteria were mixed with 900 μL of Luria Bertani media and stirred for 1 h at 37 °C and 200 rpm. From this suspension, 200 μL were seed in an LB agar plate. Then, square samples of ~1 cm^2^ were placed in the agar plate and incubated at 37 °C overnight. The inhibition distances were measured using Olympus optical microscope image analysis software (analySIS getIT, Olympus, Tokyo, Japan).

## 3. Results and Discussion

### 3.1. Structural Characterization

The structural characterization of the PLA/TiO_2_ films was done by X-ray diffraction. X-ray diffractograms of the samples (Figure S1) showed the diffraction maxima of the PLA at 2θ = 16.8° and 19.1° assigned to (200)/(110) and (203) reflexions. The XRD spectra of TiO_2_ NPs and the corresponding PLA/TiO_2_ nanocomposites showed diffraction peaks that can be assigned to both polymorphs of TiO_2_, anatase (JCPDS 89-4921) and rutile (JCPDS 89-4920) [20]. In Table S1, the assignment of XRD peaks of both polymorphs of TiO_2_ is given. From XRD patterns, it can be concluded that the structure of PLA was not affected by the presence of titania nanoparticles.

**Figure 2:**
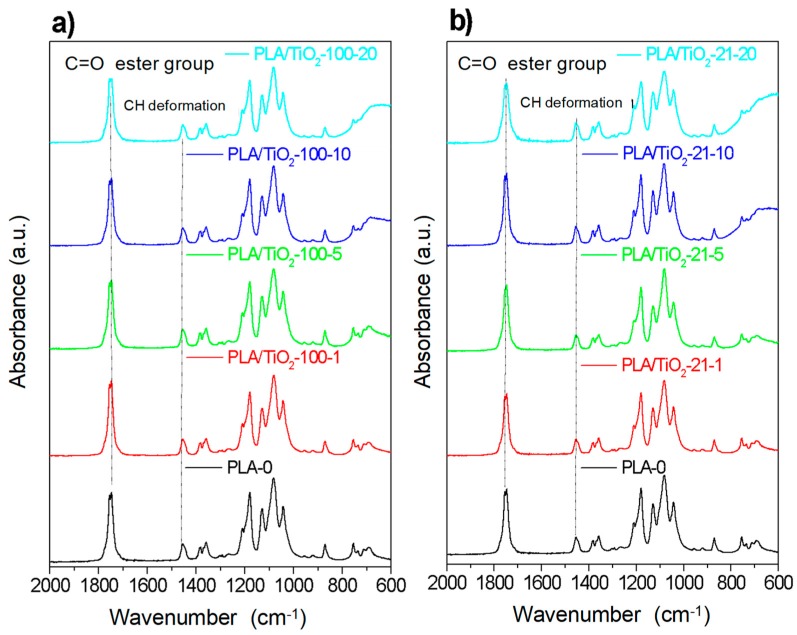
ATR-FTIR spectra for the samples with TiO_2_-100 nm (**a**) and TiO_2_-21 nm (**b**) as a function of the content of nanoparticles.

In Figure 2 are shown the ATR-FTIR spectra (mid-infrared) corresponding to PLA-0 and the different PLA/TiO_2_ composites as a function of particle content for PLA/TiO_2_ filled with the two types of nanoparticles.

In the region between 600 and 800 cm^−1^, it is possible to observe the peak due to the absorption bands corresponding to the stretching vibrations of Ti–O and Ti–O–Ti (TiO_2_) [26,27] which increases with the content of TiO_2_ nanoparticles. The typical bands of PLA appear also in this region: Namely, carbonyl groups, C=O, at 1755 cm^−1^ and bending of –CH_3_ (antisymmetric at 1454 cm^−1^ and symmetric at 1361 cm^−1^) [28]. Likewise, the stretching vibrations of C–O groups are at 1225 cm^−1^ (symmetric) and at 1090 cm^−1^ (antisymmetric). Focusing on the polymer bands with smaller intensities, it is possible to identify at 920 and 956 cm^−1^ those corresponding to the main chain vibrations (rocking of CH_3_), and also at 871 and 756 cm^−1^, which can be assigned to the amorphous and crystalline phases of PLA, respectively. Furthermore, there are no significant variations in terms of intensity or band shifting as a function of nanoparticles content and/or size for the processing conditions used. The detailed band assignment for the most representative vibrations is given in Table S2. Additionally, a full characterization of the PLA spectra in NIR region is described in Reference [22].

### 3.2. Thermal Characterization

To investigate the effect of TiO_2_ nanoparticles in the thermal properties of the materials, DSC experiments were done. Thermal properties were obtained from the second heating scan (the first heating scan was done to erase thermal history of the materials and it is not included here for discussion). The characteristic temperatures corresponding to the different transitions measured, glass transition, crystallization, and melting, are collected in Table S3.

Results showed that the main transitions associated to the typical thermal behavior of the pure PLA were present. Glass transition temperature was observed at T_g_ = 64–65 °C, similar to literature values [29,30]. Cold crystallization of the sample was present in all the samples during the heating scan with a peak temperature, T_c_ at ~137 °C. Melting process was observed as an endothermic peak with a melting temperature, T_m_, at approximately 167 °C. Considering the data collected in Table S3 (see Supplementary Material), it can be observed that the addition of TiO_2_ nanoparticles to the polymer matrix did not produce significant changes in the characteristic temperatures of the PLA matrix, indicating that TiO_2_ NPs caused little effect on the dynamics of the macromolecular chains in the samples under study, producing similar effects on the crystalline regions of the nanocomposites samples under the same processing conditions [7,31,32]. On the other hand, the composite samples of PLA/TiO_2_ crystallize in a temperature range of 100–130 °C, which would correspond mainly to an ordered crystalline α-phase structure [32].

**Figure 3:**
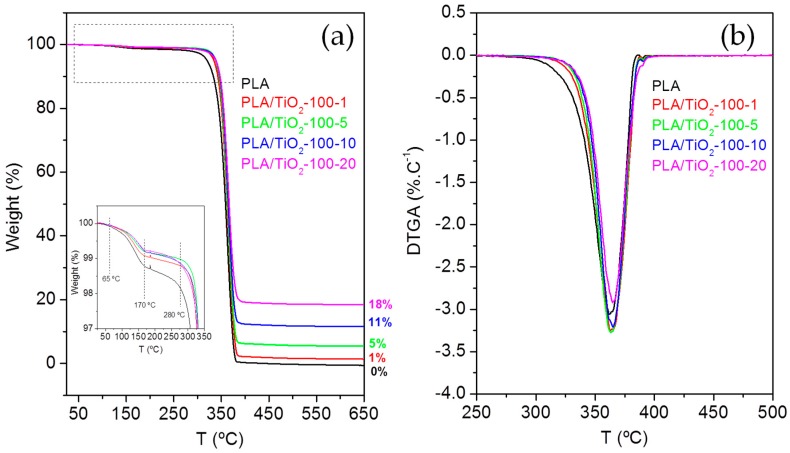
(**a**) Thermogravimetric analysis curve and (**b**) Differential thermogravimetric Analysis, DTGA, curve for the composites based on PLA/TiO_2_-100.

Thermal characterization of the samples was completed by thermogravimetric analysis (TGA), to determine the degradation temperatures of the different materials. Figure 3a illustrates the weight loss, as a percentage, as a function of heating temperature and Figure 3b the first derivative of the weight loss as a function of temperature (differential thermogravimetric analysis, DTGA curve) for the PLA/TiO_2_ nanocomposites filled with titania particles of < 100 nm. Similar plots were obtained for PLA/TiO_2_ systems filled with TiO_2_ NPs of 21 nm (Figure S2).

In all the samples, similar TGA curves were observed (Figure 3a). First, there is an initial mass loss between 65–170 °C, attributed to the loss of water from moisture. After that, a significant mass loss between 280–390 °C is observed, which corresponds to the decomposition of the PLA. Finally, from 390 °C to 500 °C thermal analysis curves slow down to complete the decomposition of the PLA matrix [33] until a constant mass is reached. The constant mass remaining at the end of each TGA experiment (see Figure 3 for PLA/TiO_2_-100 and Figure S2 for PLA/TiO_2_-21) corresponds to the inorganic material, i.e. the TiO_2_ NPs, which is very close to the theoretical amount of particles in the composites. 

From the DTGA (Figure 3b), the degradation temperatures corresponding to a 5% and 95% mass loss of PLA, T_5_ = 331.9 °C and T_95_ = 382.9 °C, were estimated. The DTGA curves showed that in the nanocomposite materials (both filled with TiO_2_-100 nm and TiO_2_-21 nm) the degradation begins at slightly higher temperatures, as can be confirmed by T_5_ and T_95_ (Table 1). Therefore, nanocomposites have slightly higher stability than pure PLA, i.e., their thermal degradation occurs at higher temperatures than that of pure PLA. A similar trend was observed for the temperature at which the degradation rate is maximum, the peak temperature of the DTGA curve, T_p_, which for PLA-0 sample is T_p_ = 361.8 °C, whereas for the nanocomposite samples showed higher peak values. The characteristic degradation temperatures T_5_, T_95_, and T_p_ are collected in Table 1 for all the samples under study. Previous studies have also shown slightly higher degradation temperatures of the polymer matrices when nanoparticles are present (LDPE/AgNPs) [25].

### 3.3. Antimicrobial Behaviour

#### 3.3.1. Study of Biofilm Development on the Surface of the Materials

**Figure 4:**
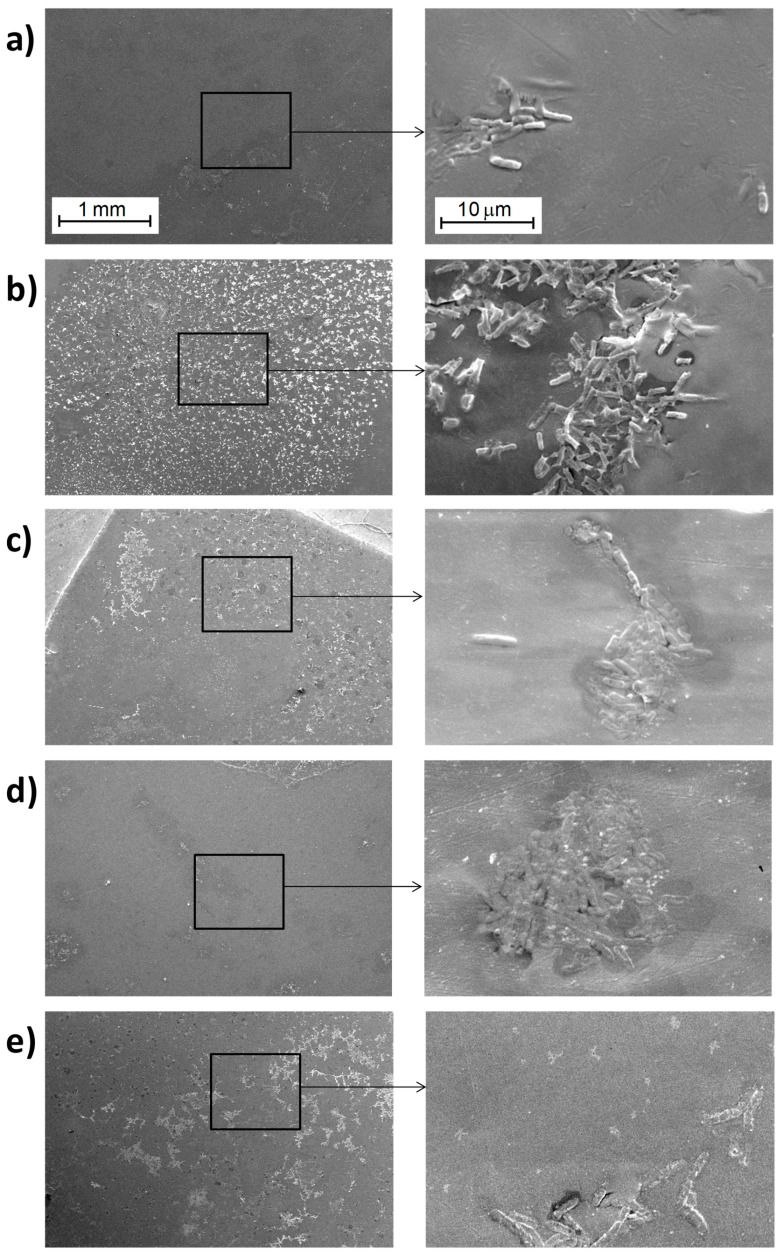
SEM micrographs for the PLA/TiO_2_-21-x system observed with the secondary electrons (SE) detector: (**a**) PLA-0, (**b**) PLA/TiO_2_-21-1, (**c**) PLA/TiO_2_-21-5, (**d**) PLA/TiO_2_-21-10 and (**e**) PLA/TiO_2_-21-20 obtained at different magnifications (left side at 50× and right side at 5000×).

**Figure 5:**
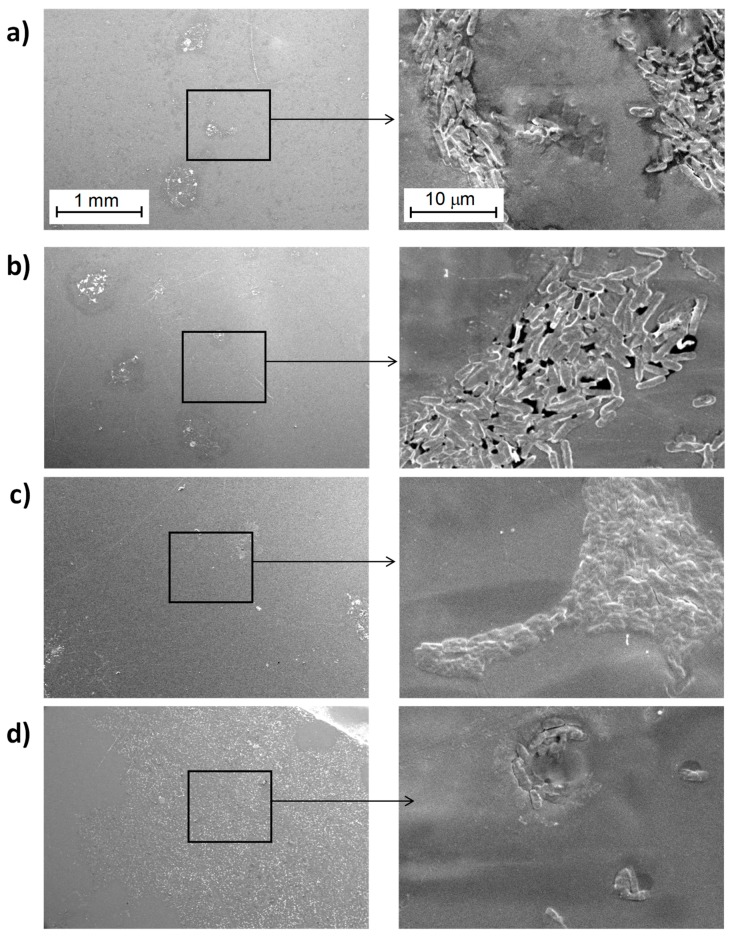
SEM images obtained with the secondary electrons (SE) detector for: (**a**) PLA/TiO_2_-100-1, (**b**) PLA/TiO_2_-100-5, (**c**) PLA/TiO_2_-100-10 and, (**d**) PLA/TiO_2_-100-20 obtained at different magnifications (left 50×, right 5000×) (Images for PLA-0 correspond to Figure 4a).

A set of micrographs obtained by SEM of the surfaces of the materials after culturing the samples in the presence of *E. coli* DH5α for the PLA/TiO_2_ nanocomposites are shown in Figure 4 and Figure 5, corresponding to materials filled with 21 nm particles (Figure 4) and with <100 nm particles (Figure 5).

**Figure 6:**
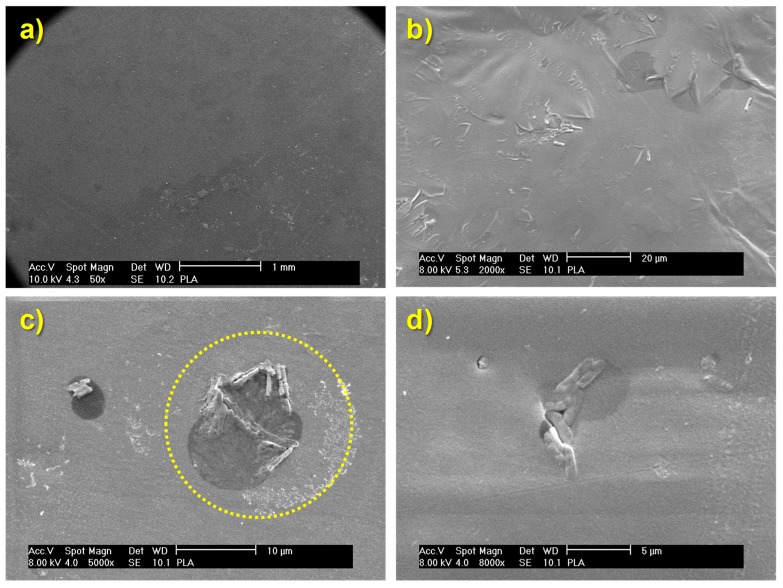
SEM micrographs of the biofilm generated on the surface of pure PLA (PLA-0) observed at different magnifications: (**a**) 50×; (**b**) 2000×; (**c**) 5000× and (**d**) 8000×.

In the case of PLA (Figure 4 and Figure 6), only some bacteria are clearly visible which could induce to think that their proliferation was minimal. However, careful observations at higher magnifications of 5000× and 8000× it is possible to identify some hollow areas where it is clearly seen that bacteria are below a material that hides them (See dashed circled area in Figure 6c). Therefore, it is possible to conclude that above pure PLA there is a larger bacteria proliferation; indeed, such proliferation rate ends up in the generation of a biofilm that has a great amount of extracellular polymeric substance, EPS [34,35,36,37], which surrounds the bacteria. For that reason, at the end, such bacteria are hidden when the samples are studied using SEM. It is important to bear in mind that SEM technique when using the backscattered electrons signal (BSE), allows the visualization of surfaces and morphologies associated to compositional changes in which elements with different atomic numbers are involved. In this study, both the bacteria and the EPS are mainly formed by carbon, and consequently they are indistinguishable by using the backscattered electron signal from a scanning electron microscope.

Regarding the results of nanocomposite materials, bacteria can be seen more easily as individual entities (Figure 4, Figure 5, Figure S3 and S4). This result may be due to a smaller amount extracellular polymeric substance (EPS) coating the biofilm formed on the surface of the nanocomposite materials. Comparing the images of the nanocomposite materials at different magnifications as a function on the content of TiO_2_ nanoparticles (Figures S3 and S4) no significant differences are seen. In some cases, it is possible to recognize more clearly the lack of continuity of the biofilm formed; but this effect may be basically due to the region selected for inspection.

**Figure 7:**
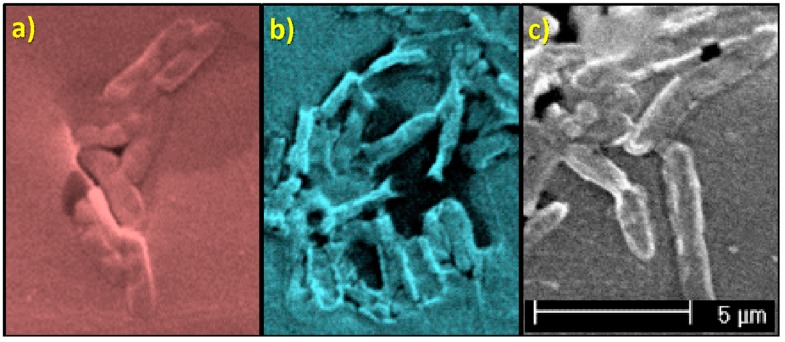
Morphology of bacteria as a function of the material on which they develop: (**a**) PLA-0; (**b**) PLA/TiO_2_ systems (φ ∼21 nm) and (**c**) PLA/TiO_2_ systems (φ < 100 nm).

In general, *E. coli* are approximately 500-600 nm width, with a length varying between 2–3 μm depending on the bipartition state in which they are found [25,38]. Slight differences among bacteria can be seen, though (Figure 7). Apparently, there are larger bacteria oblong shaped (Figure 7a) and smaller ones that tend to have a cylindrical geometry (Figure 7b) and some other bacteria are stretched and elongated (Figure 7c). These last two geometries are associated to bacteria that have grown on the surface of nanocomposite materials: The first one (Figure 7b) corresponds to bacteria grown above nanocomposites with 21 nm-sized nanoparticles, and the second geometry corresponds to bacteria grown on nanocomposites with <100 nm sized nanoparticles.

The initial conclusion that can be drawn from all these results is that the presence of TiO_2_ nanoparticles decreases bacterial growth and biofilm development of *E. coli* (DH5α) (Figure 6, Figures S3 and S4). These effects may be due to a direct interference on bacterial metabolism. The bacteria observed under the presence of nanoparticles appear to be smaller in size or elongated so it seems that their growth is altered. Moreover, bacteria grown on the nanocomposites containing particles with smaller diameter, 21 nm (Figure S3b), are even smaller compare to those grown on the composites containing the <100 nm nanoparticles. This observation may be related with the surface-to-volume ratio of the particles, which might vary the oxidative catalytic behavior of titanium oxide towards the organic material associated to both EPS and bacteria themselves.

#### 3.3.2. Kirby-Bauer Diffusion Test

**Figure 8:**
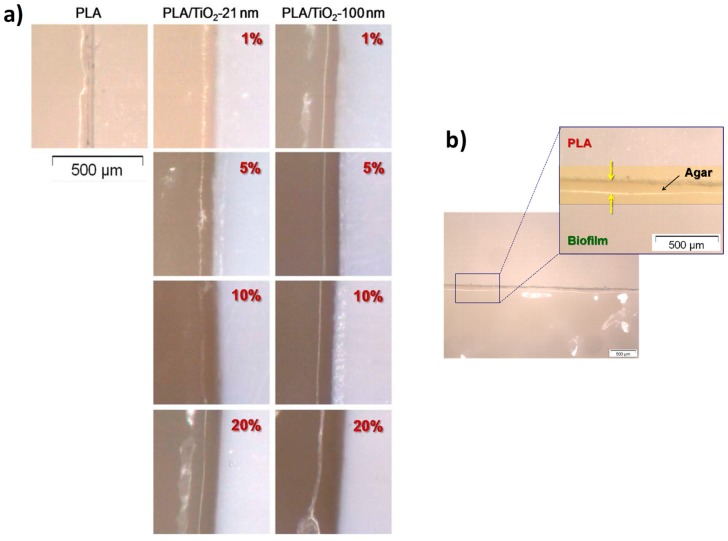
(**a**) Optical micrographs corresponding to the interphases without bacteria obtained with the Kirby-Bauer experiment; (**b**) zoomed area illustrating the measurement of the inhibition distances after the Kirby-Bauer experiment.

Figure 8 shows the optical micrographs associated to the interfaces without bacteria identified in the materials after the experiments of the modified Kirby-Bauer diffusion test. First, it should be noted that PLA itself gives rise to a small region in which there has been no bacterial growth, so it may be concluded that PLA itself has some antibacterial behavior. It can be observed that the addition of only 1% of TiO_2_ nanoparticles increases the size of this region. To gather more precise information, measurements of the inhibition distances for each PLA/TiO_2_ system were done. 

**Figure 9:**
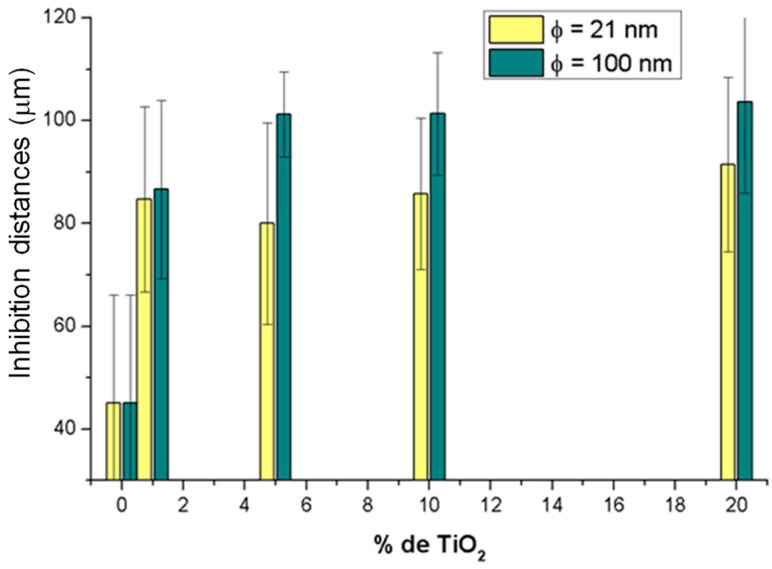
Inhibition distances calculated as a function of TiO_2_ particle size (21 nm, in yellow and <100 nm in green) and as a function of particle content (0%, 1%, 5%, 10% and 20%, wt %).

The average value of these inhibition distances calculated from the Kirby-Bauer diffusion test is represented in Figure 9 as a function of the TiO_2_ particle size and content.

Results from Figure 9 show that, when pure PLA is compared with the sample with 1% TiO_2_ nanoparticles (PLA/TiO_2_-1) the inhibition distance increases from ~45 μm to slightly less than 90 μm. Therefore, inhibition distance is almost doubled with only 1% TiO_2_ nanoparticles. As particle content in TiO_2_ nanoparticles increases from 1 to 20 wt %, average inhibition distances close to 90 μm were found, irrespective of particle content or size. While for composites with 5%, 10%, and 20% in TiO_2_ NPs it may seem that inhibition distances are longer for larger TiO_2_ nanoparticles, the variations observed in the experimental data are not high enough to consider such variations in inhibition distances to be significant, thus concluding that particle size, in these samples, do not affect inhibition distances.

## 4. Conclusions

In this work PLA/TiO_2_ nanocomposite materials were prepared and characterized to evaluate their potential uses as antibacterial materials. The variables considered for this research were particle size (21 nm and <100 nm) and particle content (0%, 1%, 5%, 10%, and 20%, wt %). The effect of particle size and content on the structural, thermal behavior and antibacterial behavior of a PLA matrix were considered. The presence of titania nanoparticles did not seem to exert high structural changes in the polymer matrix. Regarding thermal behavior, thermal transitions of the PLA matrix (glass transition, cold crystallization and melting) occurred at similar temperatures irrespective of the presence of the particles. The presence of the particles in the different PLA/TiO_2_ nanocomposites slightly increased the thermal degradation temperature of the materials as compared with pure PLA in TGA experiments.

The behavior of the materials against bacterial growth and biofilm development revealed that the presence of TiO_2_ nanoparticles considerably decreases the amount of extracellular polymeric substancd (EPS) and slightly alters the size of bacteria. Finally, in relation to the effect of particle size on the effectiveness of antibacterial action of the nanoparticles, the results obtained here from the Kirby-Bauer diffusion test were not conclusive. Therefore, future experiments should be done to clarify this issue.

## Figures and Tables

**Table 1:** Characteristic degradation temperatures for the PLA/TiO_2_ systems determined by TGA.

Sample	T_5_ (°C)	T_95_ (°C)	T_p_ (°C)
PLA-0	331.9	382.9	361.8
PLA/TiO_2_-100-1	337.4	385.9	363.0
PLA/TiO_2_-100-5	338.9	385.1	363.1
PLA/TiO_2_-100-10	341.2	384.6	364.9
PLA/TiO_2_-100-20	341.2	385.9	364.7
PLA/TiO_2_-21-1	335.8	383.3	359.5
PLA/TiO_2_-21-5	340.7	385.2	364.0
PLA/TiO_2_-21-10	341.9	385.1	366.1
PLA/TiO_2_-21-20	338.5	390.0	366.9

## Supplementary Materials

The following are available online at http://www.mdpi.com/2073-4360/10/12/1365/s1, Figure S1: X-Ray Diffraction patterns for the samples with TiO_2_-100 nm (left) and TiO_2_-21nm (right) as a function of the content in TiO_2_ nanoparticles: (a) PLA-0; (b) PLA/TiO_2_-1; (c) PLA/TiO_2_-5; (d) PLA/TiO_2_-10; (e) PLA/TiO_2_-20 and (f) pure TiO_2_-100 nm (left) or pure TiO_2_-21 nm (right), Figure S2: (a) Thermogravimetric analysis curve and (b) DTGA curve for the systems based on PLA/TiO_2_-21, Figure S3: From top to bottom: Each row corresponds to SEM micrographs obtained at different magnifications: 50×; 2000×; 5000× and 8000× PLA/TiO_2_ nanocomposite materials (φ ∼ 21 nm) as a function of the content in TiO_2_ nanoparticles in each column: (a) 1%; (b) 5%; (c) 10% and (d) 20% (wt %), Figure S4: From top to bottom: Each row corresponds to SEM micrographs obtained at different magnifications: 50 ×; 2000 ×; 5000 × and 8000 × PLA/TiO_2_ nanocomposite materials (φ < 100 nm) as a function of the content in TiO_2_ nanoparticles in each column: (a) 1%; (b) 5%; (c) 10% and (d) 20% (wt %), Table S1: XRD peaks of both polymorphs of TiO_2_, Table S2: Band assignment for the PLA infrared spectrum in the MID-IR region, Table S3: Characteristic transition temperatures (T_g_, T_c_ and T_m_) obtained from the second heating scan in DSC experiments.
