# Supplementary material for: Preparation and Characterization of Polymer Composite Materials Based on PLA/TiO2 for Antibacterial Packaging
Source: Polymers (Basel). 2018 Dec 9;10(12):1365. doi: 10.3390/polym10121365 (PMC6401929; doi:10.3390/polym10121365)
Supplement: Supplementary file 1 [file polymers-10-01365-s001.pdf]

Electronic Supplementary Material

Preparation and Characterization of Polymer Composite Materials Based on PLA/TiO_2 for Antibacterial Packaging

Edwin A. Segura González [1,2], Dania Olmos [2,*], Miguel Ángel Lorente [2], Itziar Vélaz [3] and Javier González-Benito [2,*]

[1] Dirección de Investigación, Universidad Interamericana de Panamá, Ciudad de Panamá, Panamá; edwin_segura@uip.edu.pa

[2] Department of Materials Science and Engineering and Chemical Engineering, Instituto de Química y Materiales Álvaro Alonso Barba (IQMAA), Universidad Carlos III de Madrid, Leganés 28911, Madrid, Spain; malorente@ing.uc3m.es

[3] Departamento de Química, Facultad de Ciencias, Universidad de Navarra, 31080 Pamplona, Navarra, Spain; itzvelaz@unav.es

* Correspondence: dolmos@ing.uc3m.es (D.O.); javid@ing.uc3m.es (J.G.-B.);
Tel.: +34-916249447 (D.O.); +34-916248870 (J.G.-B.)

Received: date; Accepted: date; Published: date

3. Results and discussion

3.1. Structural characterization.

<u>*X-Ray Diffraction*</u>

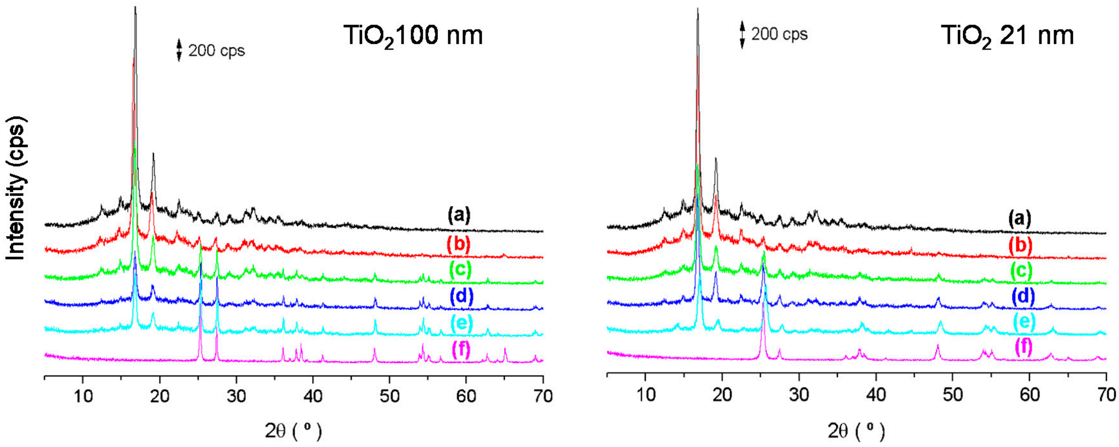

Figure S1.-X-Ray Diffraction patterns for the samples with TiO_2-100nm (left) and TiO_2-21nm (right) as a function of the content in TiO_2 nanoparticles: a) PLA-0; b) PLA/TiO_2-1; c) PLA/TiO_2-5; d) PLA/TiO_2-10; e) PLA/TiO_2-20 and f) pure TiO_2-100nm (left) or pure TiO_2-21 nm (right).

In both plots, the characteristic peaks of PLA-0 and TiO_2 were observed. Crystalline peaks in the nanocomposite materials associated with PLA appear in the interval of 2Θ = 15-25°. As the content of nanoparticles increases in the polymer matrix, the appearance of peaks from TiO_2 can

be observed. The peaks associated to TiO_2 showed lines that belonged to both anatase and rutile (see **Table S1**).

Table S1. XRD peaks of both polymorphs of TiO_2.

TiO_2 (Rutile)		TiO_2 (Anatase)	
**2θ (°)**	**(hkl)**	**2θ (°)**	**(hkl)**
27.4	110	25.3	101
36.1	101	36.9	112
41.2	111	37.8	004
54.3	211	38.4	112
56.6	220	48.0	200
64.0	310	53.9	105
65.0	221	55.0	211
		62.7	215/204
		69	116

<u>*Fourier Transformed Infrared Spectroscopy (FTIR)*</u>

Table S2. Band assignment for the PLA infrared spectrum in the MID-IR region [1,2]

Frequency (cm^{-1})	Band assignment	Frequency (cm^{-1})	Band assignment
2997	$\upsilon_{as}(CH_3)$	1209	$\upsilon_{as}(COC) + \rho_{as}(CH_3)$
2945	$\upsilon_{s}(CH_3)$	1180	◎$_{as}$COC + ρ_{as}CH$_3$)
2920	◎$_{as}$(CH$_2$)	1128	$\rho_{as}(CH_3)$
2881	◎(CH$_3$)	1082	◎$_{s}$(COC)
2850	◎$_{s}$(CH$_2$)	1043	◎(C-CH$_3$)
1747	◎(C=O)	957	CH$_3$ + ◎CC
1452	$\delta_{as}(CH_3)$	918	ρ(CH$_3$ + ◎CC)
1381	$\delta_{s}(CH_3)$	868	◎(C-COO)
1359	$\delta_{as}(CH_3)$	756	δ(C=O)
1304	δ(CH)	700	γ(C=O)
1267	◎$_{as}$(COC) + δ(CH)		

[1]Auras R et al. Macromol Biosci 2004;4: 835–64. [2]Krikorian V, Pochan DJ. Macromolecules 2005; 38: 6520–7.

3.2. Thermal characterization.

Differential Scanning Calorimetry (DSC)

Table S3. Characteristic transition temperatures (T_g, T_c and T_m) obtained from the second heating scan in DSC experiments.

Sample	T_g (°C)	T_c (°C)	T_m (°C)
PLA-0	64.6	136.9	166.8
PLA/TiO_2-100-1	64.5	137.5	167.4
PLA/TiO_2-100-5	64.2	137.2	167.1
PLA/TiO_2-100-10	64.5	136.7	166.7
PLA/TiO_2-100-20	64.5	136.9	167.7
PLA/TiO_2-21-1	64.6	136.9	167.8
PLA/TiO_2-21-5	64.6	136.9	166.5
PLA/TiO_2-21-10	64.8	137.4	167.9
PLA/TiO_2-21-20	65.3	136.3	167.4

Thermogravimetry Analysis (TGA)

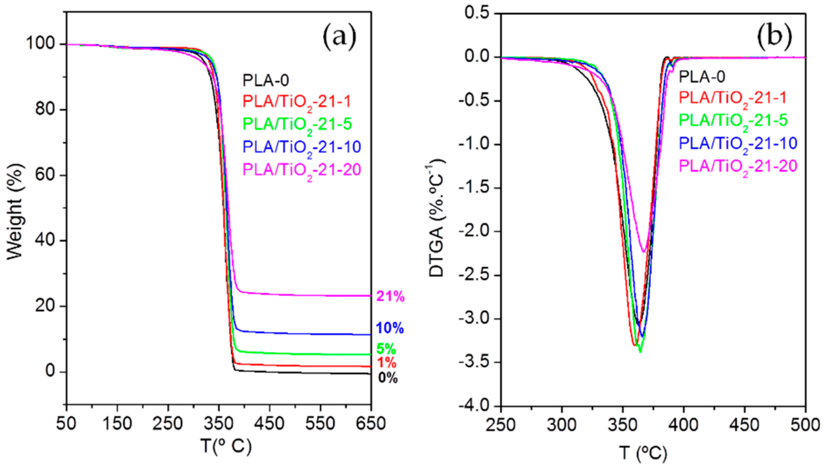

Figure S2. (a) Thermogravimetric analysis curve and (b) DTGA curve for the systems based on PLA/TiO_2-21.

3.3. Antimicrobial behaviour.

3.3.1. Study of biofilm development on the surface of the materials

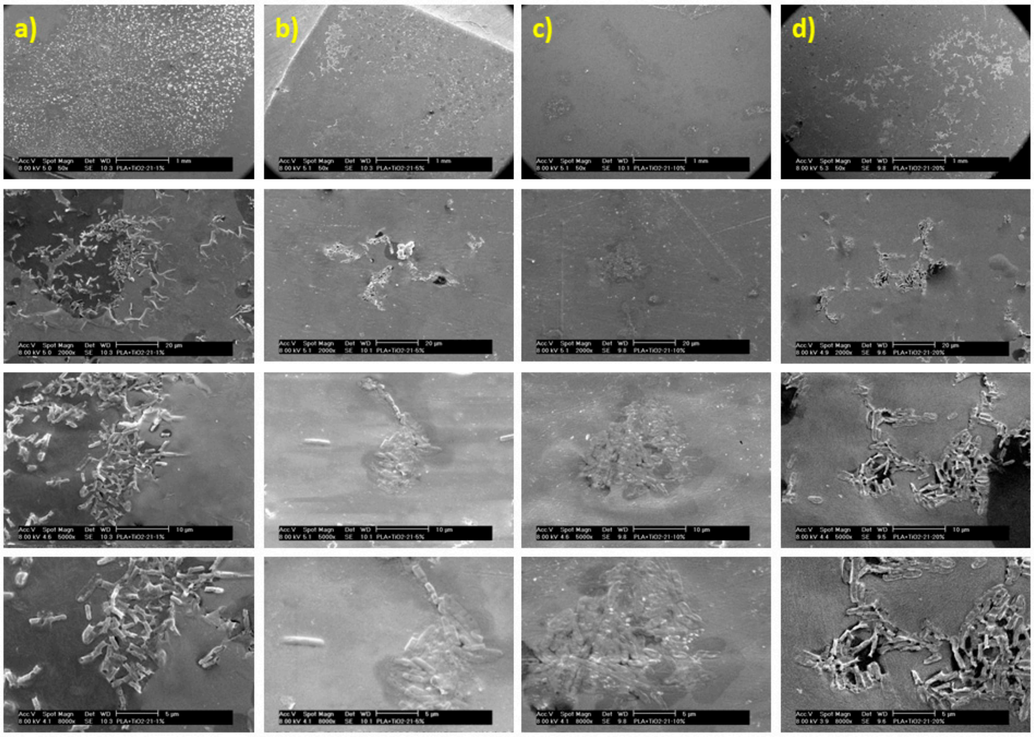

Figure S3. From top to bottom: Each row corresponds to SEM micrographs obtained at different magnifications: 50 ×; 2000 ×; 5000 × and 8000 × PLA/TiO_2 nanocomposite materials (ϕ ~ 21 nm) as a function of the content in TiO_2 nanoparticles in each column: a) 1%; b) 5%; c) 10% and d) 20% (wt%).

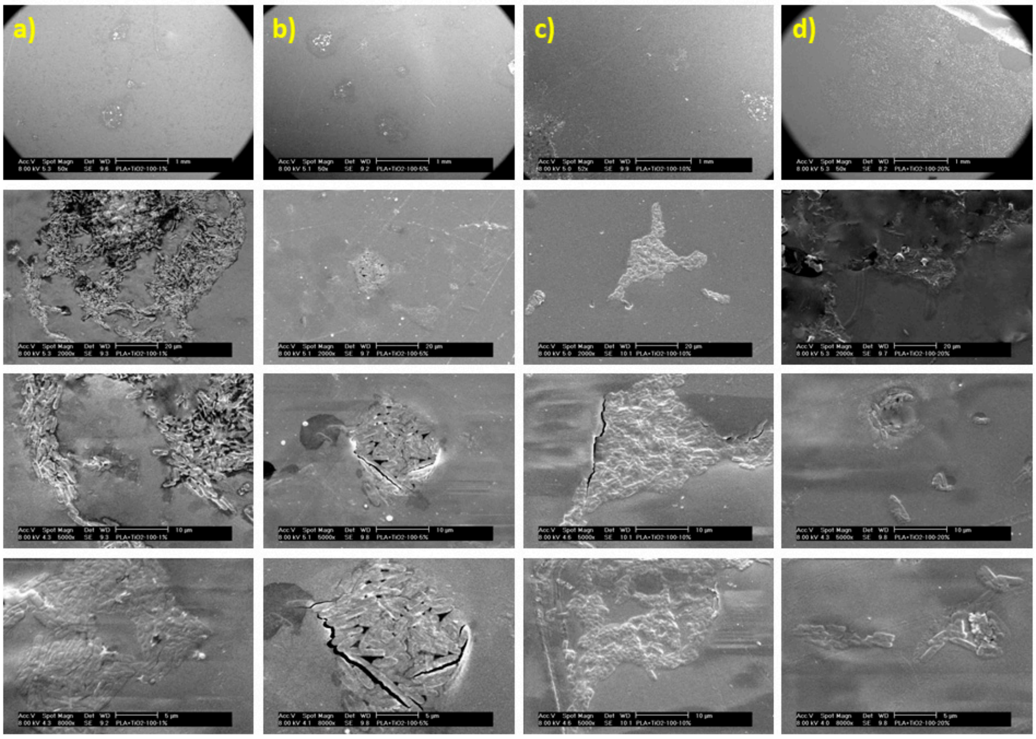

Figure S4. From top to bottom: Each row corresponds to SEM micrographs obtained at different magnifications: 50 ×; 2000 ×; 5000 × and 8000 × PLA/TiO_2 nanocomposite materials (ϕ < 100 nm) as a function of the content in TiO_2 nanoparticles in each column: a) 1%; b) 5%; c) 10% and d) 20% (wt%).
